# HIV/AIDS vaccines for Africa: scientific opportunities, challenges and strategies

**DOI:** 10.11604/pamj.2015.20.386.4660

**Published:** 2015-04-20

**Authors:** Nyasha Chin'ombe, Vurayai Ruhanya

**Affiliations:** 1 Department of Medical Microbiology, College of Health Sciences, University of Zimbabwe, P O Box A178, Avondale, Harare, Zimbabwe

**Keywords:** HIV/AIDS, vaccines, Africa

## Abstract

More than decades have already elapsed since human immunodeficiency virus (HIV) was identified as the causative agent of acquired immunodeficiency syndrome (AIDS). The HIV has since spread to all parts of the world with devastating effects. In sub-saharan Africa, the HIV/AIDS epidemic has reached unprecedented proportions. Safe, effective and affordable HIV/AIDS vaccines for Africans are therefore urgently needed to contain this public health problem. Although, there are challenges, there are also scientific opportunities and strategies that can be exploited in the development of HIV/AIDS vaccines for Africa. The recent RV144 Phase III trial in Thailand has demonstrated that it is possible to develop a vaccine that can potentially elicit modest protective immunity against HIV infection. The main objective of this review is to outline the key scientific opportunities, challenges and strategies in HIV/AIDS vaccine development in Africa.

## Introduction

Since the human immunodeficiency virus (HIV) was previously identified as the etiologic agent for acquired immunodeficiency syndrome (AIDS) more than three decades ago, the virus has spread to almost all parts of the world [1]. Globally, more than 34 million people are infected and living with the virus [2]. In Sub-Saharan Africa, the HIV/AIDS pandemic has reached unprecedented levels in most countries especially in Southern Africa [2]. The HIV that causes AIDS is a lentivirus and has an RNA genome that encodes nine open reading frames. The nine proteins encoded are classified into four groups, structural proteins (Gag and Env), enzyme proteins (Pol), regulatory proteins (Tat and Rev) and (d) accessory (auxiliary) proteins (Vpu, Vpr, Vif and Nef). Most of these genes are targets for the development of vaccines to be used for the prevention and control of HIV infection in Sub-Saharan Africa. The recent Thailand's RV144 phase-III trial has shown that it is possible to develop a vaccine that can induce some protective immune responses against HIV acquisition [3].

## Methods

This was not a systematic review. So medical literature related to HIV/AIDS vaccines and other aspects of HIV in Africa was searched unsystematically. From the database, PubMed, specific search approaches were used. We searched the literature using keywords such as “HIV vaccines”, “HIV immune responses”, “HIV vaccines in Africa”, “Challenges HIV vaccines”, “HIV genetic diversity” and “HIV vaccine approaches”. Only articles with data or results of interest to HIV vaccines in Africa were included. The articles selected were all in English. Most of the publications we used were from 1983 to 2013. We also collected literature related to HIV vaccines and biology of HIV infection from sources such as books and reports from organizations such as UNAIDS (the Joint United Nations Programme on HIV/AIDS) and World Health Organization.

## Current status of knowledge

### Immunological expectations of HIV/AIDS vaccines

In Sub-Saharan Africa, the main mode of HIV infection is through heterosexual transmission. It is therefore critical that HIV/AIDS vaccines for the region provoke both mucosal and systemic innate and adaptive immune responses against the virus. Immune cells of the innate system play a fundamental role in preventing or control HIV infection especially in the early stages of infection [4]. These cells secrete chemokines and cytokines that have the potential to block viral transmission and replication at mucosal sites. Natural killer (NK) cells are important cells of the innate immune system and they are capable of killing virus-infected cells either directly or through antibody-dependent cell-mediated cytotoxicity and they can produce interferon gamma (IFN-γ) and β-chemokines, which have anti-HIV activities [5]. Beta-chemokine-specific responses can also inhibit HIV entry during infection [6]. The antiviral activities of NK cells soon after SIV infection have been demonstrated in rhesus macaques [7]. Apart from NK cells, other cells of the innate system such as macrophages and dendritic cells also secrete a number of chemokines and cytokines that contribute in controlling HIV infection during the early stages [8]. In many ways, innate immune response controls the disease and regulates the nature and quality of the subsequent adaptive immune responses [9]. Thus vaccines that provoke the innate immune system may suppress HIV replication during the early stages of infection. However, one of the greatest challenges is to develop the HIV/AIDS vaccines that can activate the innate immune system, thereby subsequently signaling the adaptive arm of the system.

CD8+ T cell responses are an important arm of adaptive immunity and can play a major role in preventing or controlling of viral infections. In HIV infection, CD8+ cytotoxic T lymphocytes (CTLs) have the potential to destroy virally-infected cells by a number of ways [10]. First, the binding of HIV-specific CD8+ T cells to viral peptides presented by human leukocyte antigens (HLAs) on the surface of infected cells have the capacity to trigger lysis of HIV-infected cells [10, 11]. The lysis of the virus-infected cells occurs through the production of perforin and granzymes, which penetrate into the cells and induce apoptosis [12]. Second, HIV-specific CD8+ T cells produce cytokines such as IFN-γ and tumour necrosis factor alpha (TNF-α), which all have antiviral activities that can suppress replication of HIV [13]. Third, CD8+ T cells produce chemokines that block HIV entry by binding to HIV coreceptors [13]. The cytolytic activities of CD8+ T cells can further be enhanced by these chemokines [11, 13]. Scientific evidence are available that show that CD8+ T cell responses play an important role in controlling HIV infection or eliminating infected cells. In primary infection, HIV-specific CD8+ T-cells have been found to suppress or reduce viral replication [10, 14]. After infection, it has also been found that the emergence of HIV-specific CD8+ T-cells is always associated with a decreased viral load [15]. Potent HIV-specific CD8+ T cell responses in chronic infection are also always correlated with low viraemia and reduced disease progression [15]. In animal models of HIV infection, it has been shown that depletion of CD8+ T lymphocytes would lead to failure to control viral replication [16]. In vitro studies have further demonstrated that HIV-specific CTLs can efficiently kill HIV-infected cells and inhibit viral replication [17]. It has also been noted that highly HIV-exposed seronegative African sex-workers had detectable HIV-specific lysis [18]. The emergence of CD8+ T cell epitope escape mutants has also been shown to be associated with rapid disease progression and this remains a great challenge to vaccine development [19]. Furthermore, vaccine-induced CD8+ T cell responses have been shown to protect macaques from developing AIDS after a challenge with simian-human immunodeficiency virus [20]. These studies clearly demonstrate that protection or control of HIV infection requires CD8+ T cell responses and vaccines targeted for Africans should induce this type of immune response.

There is also mounting evidence to support that CD4+ T helper (Th) responses play critical roles in prevention or control of HIV-1 infection and replication [21]. HIV-infected individuals who are long-term non-progressors have been found to have strong CD4+ T cell responses to HIV antigens [21]. CD4+ T cell responses were also associated with control of HIV viremia as it was noted that patients with the highest CD4+ T cell responses had the lowest viral loads, whereas patients with the lowest CD4+ T cell responses had the highest viral loads [22]. In some patients, strong CD4+ T helper responses were found to be associated with strong CD8+ CTL responses [21]. Recent studies have further suggested that HIV-specific CD4+ Th1 cells producing INF-β and IL-2, together with IgG2 were important in long-term control of HIV infection and reduced viraemia [23]. Thus, CD4+ T cells provide immunological help to CD8+ T cell responses. CD4+ T cells also produce cytokines such as IFN-γ and TNF-α that have antiviral activities [24]. CD4+ T cells further provide help for antibody responses that may be critical for neutralization of the virus. There have been recent reports of the cytotoxic CD4+ T-cells detected in HIV infection [25]. Therefore, HIV/AIDS vaccines for Africa need to induce specific CD4+ T cell responses that would enhance both CD8+ T cell and humoral immune responses.

Humoral immune response that is mediated by antibodies produced by B cell lymphocytes plays a protective role against many viral pathogens including HIV [26]. The antibodies offer protection by neutralizing pathogen antigens, thereby preventing infection. Infection with HIV induces virus-specific antibody responses [26]. It has been demonstrated that HIV-infected individuals elicit high levels of antibodies against different viral antigens [27]. The antibodies that have the capacity to neutralize HIV (neutralizing antibodies) are mainly directed against the viral Env protein and they can potentially block HIV replication or infection by virus neutralization or antibody-dependent cellular cytotoxicity of HIV-infected cells [28]. Neutralizing antibodies are normally correlated with significant decline of the primary viremia [29]. Furthermore, some infected individuals with strong neutralizing antibody responses control their viraemia for a long time [30]. Studies in animal models have demonstrated that passive immunization with monoclonal antibodies generated from HIV-infected individuals such as 2F5, 2G12, and 4E10 could protect monkeys from challenge infections [31, 32]. Studies done on RV144 trial follow-up also demonstrated that antibodies to variable loops of HIV-1 Env were associated with reduction in the acquisition of the virus [3]. Non-neutralizing antibodies through antibody-dependent cell-mediated-ADCC and antibody-dependent cell-mediated viral inhibition also play a key role in preventing or controlling HIV infection [33]. Therefore, HIV/AIDS vaccines developed for Africans should be capable of eliciting both neutralizing and non-neutralizing antibodies that can prevent establishment of a new HIV infection or reduce the replication of genetically diverse HIV viruses.

Most human pathogens such as HIV infect their hosts through the mucosal surfaces. These mucosal surfaces are found in the gastro-intestinal, urogenital and respiratory tracts and play important role in the uptake and transport of pathogens or antigens [34, 35]. In Africa, the majority of HIV infections occur through mucosal exposure to seminal fluid or vaginal secretions of infected individuals. On top of mucosal transmission, HIV replicates in the mucosal lymphoid tissue before systemic spread [36]. The CD4+ T cells of mucosal lymphoid tissues are also the targets of HIV throughout infection, leading to their depletion [37]. Mucosal plasma cells synthesize secretory immunoglobulin A (IgA) that has the potential to neutralize HIV [38]. Mucosal surfaces are rich in immune cells such as dendritic cells, macrophages, CD4+ and CD8+ T cells which can play important roles in provoking immunity to a variety of pathogens including HIV [39]. Studies have demonstrated the presence of SIV- and HIV-specific CD8+ T cell responses in the genital tracts of infected macaques [40]. Inducing humoral, CD8+ and CD4+ T cell responses at mucosal surfaces with vaccines can potentially prevent or control HIV replication in the mucosal lymphoid tissue. One of the key advantages of mucosal vaccination against HIV is that mucosal immunity protects systemic infection, whereas systemic immunity poorly protects against mucosal infection [41]. The other advantage of mucosal vaccination is that antigenic exposure at one mucosal site activates B and T cells to emigrate and home to other mucosal surfaces, thereby conferring protection at these sites [42]. Protective vaccine-induced mucosal immunity against HIV has been demonstrated in animal models [43]. Therefore the challenge is to develop HIV vaccines for Africa that can induce both B and T cell responses in mucosal tissues.

### Immune correlates of HIV protection in Africa

To date, the exact immune correlates of protection against natural HIV infection are poorly defined. However, it is generally regarded that HIV protective immune responses should comprise of CD8+ T, CD4+ T cell and humoral immune responses. Different components of these immune responses have different effectiveness in preventing or controlling HIV infection. Whereas neutralizing antibodies can potentially block HIV infection, they are not effective against cells that are already infected [44]. Cellular immune responses can control HIV infection, but cannot prevent infection. The key challenge is to develop vaccines that can induce multiple forms of immune responses against HIV. Mucosal immune responses may potentially be required to control the early stages of HIV infection and replication. Mucosal immunity may also delay systemic spread of the virus. To date, we do not clearly know the exact nature, quality and magnitude of immune responses that should be elicited by the HIV or HIV/AIDS vaccine. Nevertheless, recent animal studies have suggested that vaccine-induced SIV-specific memory CD8+ T and CD4+ T cells may correlate with protection against simian AIDS disease in monkeys [45, 46]. Although CD8+ T cell and neutralizing antibodies are considered important for HIV protection or control of infection, the RV144 trial has demonstrated that non-neutralizing antibodies are also crucial [3]. Non-neutralizing antibodies can mediate antibody-dependent cellular cytotoxicity (ADCC), thereby protecting against HIV infection [47]. ADCC is important because it is associated with reduced HIV/AIDS diseases progression and prevention of cell-to-cell spread by the virus [48]. However, the rise of ADCC-escape HIV variants can be a scientific challenge to HIV/AIDS vaccine development [49]. Another key challenge is that antibodies mediated via complement system or Fc receptors can unfortunately facilitate the infectivity of the HIV [50].

### Scientific challenges to HIV/AIDS vaccine development for Africa

Current efforts in development of HIV vaccines for Sub-Saharan Africans are hampered by a number of scientific challenges. A key characteristic feature of HIV subtypes in Sub-Saharan Africa is their high genetic variability [51]. The genetic diversity and high mutation rate emanate from the failure of the HIV reverse transcriptase (RT) enzyme to proof-read the viral genome during replication [52]. The RT can further facilitate the generation of new viral genetic recombinants due to its recombinogenic properties [53]. These two factors contribute to the generation of high genetic diversity of HIV in Africa. The HIV has been divided into groups, sub-types and circulating recombinant forms (CRF) and unique recombinants [54]. Three distinct HIV-1 groups of viruses exist [54]. They are M (main), O (outlier) and N (new or non-M/non-O). Group M viruses are responsible for the majority of HIV-1 infections worldwide including Africa. Group O isolates are highly divergent from group M, their prevalence is low compared to other viruses and infection is confined to West African countries. Phylogenetic analysis of the env and gag genes of the group M has established 9 distinct subtypes (subtypes A, B, C, D, F, G, H, J and K) [55]. Different parts of Africa have different HIV-1 subtypes being prevalent. The genetic diversity of HIV-1 subtypes and emergence of new recombinants remains one of the key challenges in vaccine development in Africa. It is therefore remains a great challenge to develop HIV/AIDS vaccines that can target all the diverse HIV-1 subtypes and recombinants circulating in Africa. Another strategy will be to develop vaccines specific for specific regions of Africa that include only antigens of the circulating subtypes.

As stated above, the correlates of protection after HIV infection or vaccination are very complex and poorly understood [56]. Our poor understanding of the correlates of HIV protection makes the development of vaccines for Africans a great challenge. However, studies in Africa have given us some clues of the requirements or expectations of an AIDS vaccine. Some sex workers in Africa were found to be resistant to HIV infection despite them being exposed to the risk [18]. The high prevalence of HIV discordance in African couples also suggests that the correlates of protection exist in the African populations [57]. Further research on HIV discordance should bring a deeper understanding of some of the correlates of protection in Africans and can subsequently lead to the development of vaccines.

Currently, there are no good animal models to test HIV/AIDS vaccines. Chimpanzees and macaques are most commonly used to study the HIV pathogenesis as well as vaccines [58]. Mice are also used in pre-clinical evaluation of vaccines, but results in mice do not normally predict what will be found in humans. The use of monkey or baboon primate models in studying HIV/AIDS vaccines has also its drawbacks. Data generated from mouse, monkey or baboon models do not normally translate to what will be found in human clinical trials. Humanized mice can also be used in testing HIV/AIDS vaccines, but they do not normally elicit strong immune responses [59].

Bantu-speaking African populations of Sub-Saharan Africa are highly diverse genetically [60]. The high prevalence of high-risk exposed HIV-seronegative individuals in Africa has suggested the role of genetic factors in influencing HIV infection or immune responses to infection [61]. Some of the genetic factors include the genes of HIV coreceptors and their natural ligands, the human leukocyte antigen (HLA), apolipoprotein B mRNA-editing, enzyme-catalytic (APOBEC), tripartite motif-containing protein 5 (TRIM5) and killer cell immunoglobulin-like receptor (KIR) genes [62, 63]. The geographic and genetic variations in some of these host genes especially the HLA haplotypes in Africa are likely to affect immune responses to HIV infection or vaccinations in Africans. It is therefore important to bear this in mind when developing HIV/AIDS vaccines for Africans that genetic diversity may cause variation in immune responses.

### Scientific strategies for HIV/AIDS vaccine development for Africa

To date, there is no licensed HIV/AIDS vaccine for Africa. Several rational and empirical strategies to HIV vaccine development have so far failed dismally. However, a variety of these strategies need to be refined if we are to develop potential vaccine candidates for Africa. It has been a classical approach to use inactivated viruses as vaccines. It is possible to inactivate HIV and this strategy has been explored [64, 65]. Although the inactivated HIV vaccine candidates may be safe for use, even in immunocompromised people, the strategy is not advocated for due to poor immunogenicity elicited by these vaccines. Such HIV vaccines would not be very helpful for Africa, given their poor immunogenicity. The great challenge is to generate inactivated vaccines that are highly immunogenic in Africans. It is easy to genetically attenuate viruses such as HIV using modern tools of genetic engineering whereby mutations or deletions are introduced in specific viral genes. This generates genetically attenuated HIV vaccines, which are expected to confer immunogenicity without causing AIDS. Although some of the live attenuated HIV vaccines have shown a degree of protective efficacy in animal models this approach has not attracted much attention because of safety concerns in humans [66–68]. To date, no live attenuated HIV/AIDS vaccine candidates specifically developed for Africa have been tested in clinical trials. Subunit vaccine candidates against HIV/AIDS have been developed [69, 70]. These vaccines are based on purified HIV antigens such as Envelope and Gag. Most of the early HIV/AIDS vaccines that entered Phase I trials were based on Envelope subunit. HIV virus-like particles (VLPs) are also subunit vaccines generated from the expression of Gag alone and can be used as immunogens. They have been found to be immunogenic in animal trials especially when used in prime-boost strategies with other vaccines [70, 71]. Although HIV/AIDS subunit vaccines have been found to be immunogenic in animals, human trials have shown disappointing results.

Recombinant plasmids, when used as DNA vaccines induce immune responses specific to the antigen genes carried [72]. Most HIV DNA vaccines have been shown to be safe and to induce protective immune responses in animal models [73, 74]. Induction of both CD8+ CTL and humoral immune responses were demonstrated in animals primed with gp120 DNA vaccine and boosted with gp120 subunit vaccine [75]. A number of candidate DNA vaccines for HIV-1 have already been developed and some tested in Africa for immunogenicity [76–78]. Such vaccines are likely to be useful in Africa since they induce strong immune responses especially if they are used in prime-boost strategies. Recombinant live viruses can be exploited as vaccine vectors for heterologous antigens [79, 80]. The key advantage of viral vectors is that they can generate very strong antigen-specific CD8+ and CD4+ T cell as well as humoral immune responses [79–81]. Most viral vectors can target both the innate and adaptive immune responses at both the mucosal and systemic compartments. A number of viral vaccine vectors for HIV antigens have so far been developed and tested. These viral vaccine vectors have been genetically engineered to express different HIV antigens. Effective anti-HIV immunity, sometimes protective, has been observed in a number of animal studies in which vectors such as adenovirus, alphavirus, sendai virus, herpes simplex virus, human rhinovirus and polio virus were used to express HIV antigens [82–86]. Recombinant viral vectors therefore seem to offer great opportunities for vaccine development for Africans because of their ability to induce strong HIV-specific immune responses. Recombinant bacteria can also be used to deliver heterologous antigens to the host's immune system [87]. Their potential use as candidate HIV vaccine vectors to deliver either HIV antigens or HIV DNA vaccines is currently being increasingly studied. Recombinant Bacillus Calmette-Guerin (BCG) expressing HIV antigens has been shown to induce antigen-specific immune responses in vaccinated animals [88]. BCG is generally a good vaccine vector for HIV/AIDS because of a number of reasons such as its known safety record [89]. Another attractive bacterial vaccine vector for HIV/AIDS is *Listeria monocytogenes* [90]. The key advantage of *Listeria* as a vaccine vector is that it replicates in the cytosol, thereby inducing both strong CD8+ and CD4+ T cell responses. *Shigella* is also an attractive vector that is capable of replicating in the cytosol thereby inducing strong cellular immune responses [91]. Attenuated *Shigella* strains have already been successfully used to deliver HIV DNA vaccines, resulting in induction of HIV-specific CD8+ T responses [92]. Recombinant *Salmonella* has also a great potential as a vaccine vector for HIV [93–95]. Therefore, recombinant bacterial vaccine vectors can be harnessed in the development of HIV/AIDS vaccines for Africa.

Candidate HIV/AIDS vaccines for Africa can be used in prime-boost strategies in order to improve their potency and effectiveness. In these strategies, one vaccine is used to prime the immune system and the second vaccine is used to boost the response [96, 97]. A number of candidate HIV/AIDS vaccines have already been tested in a prime-boost strategy and have been shown to induce good responses [98, 99]. DNA vaccines have already been shown to be best at priming and recombinant viral vectors such as poxvirus vectors are good at boosting the immune responses [100]. Even the Thailand's RV144 phase-III trial used the prime-boost strategy in which a recombinant canarypox vector-based candidate was used in combination with an engineered HIV-1 gp120 protein [3].

## Conclusion

The HIV/AIDS remains a serious public health problem in Africa and development of safe, effective and affordable vaccines for the region remains a daunting challenge. However, a number of efforts have already been done to develop candidate vaccines.
